# Effectiveness and implementation of an online intervention (MINDxYOU) for reducing stress and promote mental health among healthcare workers in Spain: a study protocol for a stepped-wedge cluster randomized trial

**DOI:** 10.1186/s12912-022-01089-5

**Published:** 2022-11-10

**Authors:** Yolanda López-del-Hoyo, Selene Fernández-Martínez, Adrián Pérez-Aranda, Alberto Barceló-Soler, Jose Guzman-Parra, Esperanza Varela-Moreno, Daniel Campos, Alicia Monreal-Bartolomé, María Beltrán-Ruiz, Berta Moreno-Küstner, Fermín Mayoral-Cleries, Javier García-Campayo

**Affiliations:** 1 Institute of Health Research of Aragon (IIS Aragón), Miguel Servet University Hospital, Saragossa, Spain; 2 Departamento de Psicología Y Sociología, Facultad de Ciencias Humanas Y de La Educación, Universidad de Zaragoza, Saragossa/Huesca, Spain; 3 Research Network On Chronicity, Primary Care and Health Promotion (RICAPPS) RD21/0016/0005, Saragossa, Spain; 4 Department of Basic, Developmental and Educational Psychology, Autonomous University of Barcelona, Cerdanyola Del Vallès, Spain; 5 Navarra Medical Research Institute (IdiSNA), Pamplona, Spain; 6 Mental Health Clinical Management Unit, Regional University Hospital of Malaga, Málaga, Spain; 7 Malaga Biomedical Research Institute (IBIMA), Málaga, Spain; 8 Department of Personality, Evaluation and Psychological Treatment, Faculty of Psychology, University of Malaga, Málaga, Spain; 9 MARISTAN Network, Málaga, Spain; 10 Department of Psychiatry, Faculty of Medicine, University of Zaragoza, Saragossa, Spain

**Keywords:** Healthcare workers, Stress, EHealth, Mindfulness, Compassion, Acceptance, Implementation, Cost-utility, Cluster randomized trial

## Abstract

**Background:**

The World Health Organization has formally recognized that healthcare professionals are at risk of developing mental health problems; finding ways to reduce their stress is mandatory to improve both their quality of life and, indirectly, their job performance. In recent years, particularly since the COVID-19 pandemic outbreak, there has been a proliferation of online interventions with promising results. The purpose of the present study is twofold: to test the effectiveness of an online, self-guided intervention, MINDxYOU, to reduce the stress levels of healthcare workers; and to conduct an implementation study of this intervention. Additionally, an economic evaluation of the intervention will be conducted.

**Methods:**

The current study has a hybrid effectiveness-implementation type 2 design. A stepped wedge cluster randomized trial design will be used, with a cohort of 180 healthcare workers recruited in two Spanish provinces (Malaga and Zaragoza). The recruitment stage will commence in October 2022. Frontline health workers who provide direct care to people in a hospital, primary care center, or nursing home setting in both regions will participate. The effectiveness of the intervention will be studied, with perceived stress as the main outcome (Perceived Stress Scale), while other psychopathological symptoms and process variables (e.g., mindfulness, compassion, resilience, and psychological flexibility) will be also assessed as secondary outcomes. The implementation study will include analysis of feasibility, acceptability, adoption, appropriateness, fidelity, penetration, and sustainability. The incremental costs and benefits, in terms of quality-adjusted life years, will be examined by means of cost-utility and cost-effectiveness analyses.

**Discussion:**

MINDxYOU is designed to reduce healthcare workers’ stress levels through the practice of mindfulness, acceptance, and compassion, with a special focus on how to apply these skills to healthy habits and considering the particular stressors that these professionals face on a daily basis. The present study will show how implementation studies are useful for establishing the framework in which to address barriers to and promote facilitators for acceptability, appropriateness, adoption, feasibility, fidelity, penetration, and sustainability of online interventions. The ultimate goal is to reduce the research-to-practice gap.

**Trial registration:**

This study was registered in ClinicalTrials.gov on 29/06/2022; registration number: NCT05436717.

## Background

Although stress levels among healthcare workers are normally high, they have been accentuated by the COVID-19 pandemic. Over the last two years, healthcare workers have had to deal with the same pandemic-related stressors as any other citizens, in addition to their specific job-related stressors: continued exposure to infection, longer shifts––which has led to inevitable physical exhaustion and neglect of important aspects related to a healthy lifestyle––and difficult decisions regarding how to prioritize available resources in order to treat their patients, among others [1, 2]. Subjection to this sustained pressure, which is added to the normally high care burden of healthcare workers, entails a risk factor for developing mental health problems, including anxiety disorders, depression, somatoform disorders, post-traumatic stress disorders, and even suicidal ideation [3, 4].

The World Health Organization (WHO) has formally recognized that healthcare professionals are at risk of developing psychopathological symptoms, particularly those working in public health, primary care, emergency departments, and intensive care units [5, 6], including personnel employed by nursing homes [7]. It is essential to take care of the mental health of healthcare workers, not only to improve their quality of life, but also because the success of their work largely depends on it. Hence, it is especially necessary in the current circumstances to offer these workers effective and highly adaptive strategies that will enable them to cope with the numerous stressors to which they are exposed in order to prevent the appearance of mental health problems, also in the long term.

Many psychotherapeutic programs have been developed to combat stress. Interventions based on cognitive behavioral therapy (CBT) have proven effective for reducing stress, anxiety, and depression in different clinical populations [8–10]. Similarly, so-called “third wave” psychotherapies, such as mindfulness-based interventions (MBIs), compassion-based programs, and acceptance and commitment therapy (ACT), have also proved effective for reducing different psychopathological symptoms [11–15], including stress in healthcare professionals [16–21]. The most common format for the application of these psychotherapies has been face-to-face, both in group and individual therapy. However, for a few years, and particularly because of the pandemic, online psychotherapeutic programs have proliferated, making it possible to overcome obstacles to face-to-face attendance. They are very well suited to the needs of healthcare workers with schedules that are often difficult to reconcile [22].

There are a good number of online interventions (e.g., web-based, mobile applications, videoconference-delivered interventions, among others) aimed at treating stress, anxiety, depression, and insomnia, among others. Different studies have been published on the efficacy of online interventions to improve the quality of life and reduce the stress of healthcare workers. Some have verified that online CBT-based interventions are effective for reducing stress, suicidal ideation, or emotional distress in this population [20, 23–26], and others have also studied the efficacy of “third wave” online psychotherapies with positive results [27–30]. However, most of these programs have only achieved small effect sizes in terms of their effectiveness, and a fact that cannot be overlooked is that studies have also been published regarding online interventions found to be ineffective for reducing stress of healthcare workers, which makes further study into the characteristics of these programs and their possible limitations necessary. Moreover, the interventions that are tested in effectiveness studies are mostly discontinued once the study ends, which poses a major problem when it comes to the translation of efficacy studies on evidence-based interventions to daily clinical practice, a period estimated to be 17–20 years [31].

## Methods

### Study aims

This study aims to evaluate the effectiveness of a self-guided online program, MINDxYOU, based on “third wave” psychotherapy principles, for reducing the stress levels of healthcare workers through the practice of different exercises that promote mindfulness, acceptance, and self-compassion, in addition to indications on how to apply these skills to a healthy lifestyle. Furthermore, this project intends to emphasize the implementation of the intervention; implementation science is a discipline that is developing methods and procedures to ensure that evidence-based interventions are incorporated more quickly into daily clinical practice [32]. The implementation study includes 3 types of evaluation: 1) evaluation of the process (characteristics of the use of the intervention; measures before, during, and after implementation); 2) formative evaluation (adaptations for improvement based on participants’ feedback); and 3) summative evaluation (a compilation of the impact of the implementation strategy, evaluating the impact on indicators such as increase in the use of the intervention in the target context and economic impact).

As secondary aims, the impact of the intervention on other health-related outcomes such as psychopathological symptoms (depression, anxiety, and others) and process variables (resilience, mindfulness, compassion, and psychological flexibility) will also be evaluated, and an economic evaluation will be conducted.

### Study design

**Fig. 1:**
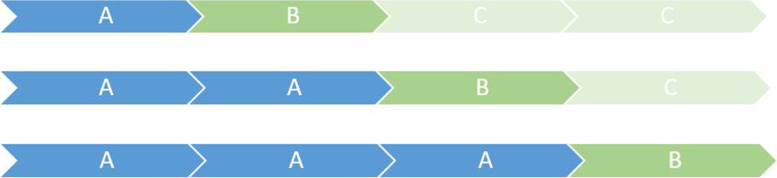
The three possible sequences of the study phases. *Note:* A = Control phase; B = Intervention phase; C = Maintenance phase. Each cell represents a period of 8 weeks

An effectiveness-implementation type 2 design will be used [33], since this study will assess both the effectiveness of the intervention and the implementation process in the same degree, as co-primary aims [34]. The empirical study will take a stepped wedge (SW) cluster randomized controlled trial design; this design implies that every participant starts the trial in the control phase (no exposure to the intervention), and then undertakes the intervention in a staggered and sequential way. Once it is completed (8 weeks), the maintenance phase takes place, during which the participant can continue to use the program at will, but without supervision. There will be three possible sequences (see Fig. 1), and each of the participating centers will be randomly assigned to one of them. The participants from each site will all follow the same sequence.

SW designs are particularly recommended when the intervention being tested has strong evidence of positive effects or is very unlikely to cause harm [35], which is the case of our study. The present work is considered a closed cohort study, since all the study participants will start at the same time and will be evaluated at different time-points to be defined beforehand; the inclusion of new participants will not be allowed once the trial has commenced.

### Participants

The study sample will comprise healthcare workers currently working in clinical settings: a hospital, a primary care (PC) center, and a nursing home in the provinces of Zaragoza (Aragon) and Malaga (Andalusia), Spain. The following specific inclusion criteria will apply: 1) employment as a physician, nurse, physiotherapist, psychologist, nursing assistant, ambulance technician; a trainee student in any health profession; or employment in a nursing home assisting patients; 2) aged between 18 and 70; 3) ability to understand Spanish; 4) digital literacy and access to a smartphone, tablet, or personal computer with Internet connection; 5) prospects of continued employment at the same site for the following 6 months; and 6) provision of informed consent.

The following exclusion criteria will apply: 1) presenting with a disorder that affects the central nervous system; 2) diagnosis of a severe mental illness (including severe depressive disorders, suicidal tendencies, bipolar disorders, panic disorders, anxiety or stress-related disorders, obsessive–compulsive disorders, and substance-related disorders); 3) presenting with a medical, infectious, or degenerative illness that is not under control; and 4) expertise in “third wave” psychotherapies (i.e., meditation, mindfulness, etc.).

### Sample size

The sample size was calculated following the recommendations for SW designs [36]. First, the sample size for individual randomization needed to be determined; in this case, with an expected effect size of *d* = 0.50 for the primary outcome (Perceived Stress Scale, PSS) [37], which is a continuous variable, a statistical power of 80%, and an alpha level of 0.050, the required sample size would be 126 participants (N_1_). Subsequently, the following formula was considered:

$$m=\frac{\mathrm{N}1 (1-p)}{\mathrm{K}-\mathrm{N}1\mathrm{p}}$$

where *m* is the necessary sample for each cluster and for each assessment moment (variable to be determined), N_1_ is the already calculated sample size (126), *p* is the intra-class correlation coefficient (assumed: 0.04), and *K* is the number of clusters (6). The result would be 24 participants per cluster, and assuming 20% attrition, 30 participants should be recruited from each site. This would imply a total sample of 180 participants.

### Procedure

The strategy to recruit study participants will commence in October 2022. It will include indirect techniques such as informative posters in the selected hospitals/PC centers/nursing homes containing a brief explanation of the study and the website link; in addition, the occupational health service of the hospital and a member of staff at each site will contact their peers and send them an informative email with a brief explanation of the study and the website link. Consequently, any individuals interested in taking part in the study will find on the website all the details regarding the study procedures and aims, together with the rights of every participant and an explanation of the way in which their data will be processed.

Informed consent will then be documented via electronic signature, and complying candidates will undergo a basic screening process, which will consist of a checklist of the inclusion criteria. Candidates who meet these self-reported criteria will be presented with a schedule for the following 2 weeks and they will be asked to select a time for a 20-min video call or telephone call from a member of the research team who will conduct the complete screening of the participant, including the assessment of psychiatric diagnoses (MINI v7.0.2). Candidates who meet all the criteria will formally be included as participants in the study and will be offered information regarding the following procedures, which will start with a baseline evaluation including different self-reported questionnaires that the participant will complete using an online platform. The participant will receive via email or text message the link to the evaluation platform. Once the control phase is over, the participant will receive via email or text message their credentials (i.e., user and password) to access to the online intervention platform so they can start the MINDxYOU program. An external researcher, not related to this study, will conduct the randomization process to one of the three possible sequences using EPIDAT 4.2 (see Fig. 1).

Clear indications will be given on how to use the platform in the welcome module (see Table 1). Participants will be assessed for all the study variables every 8 weeks; the usability measure (System Usability Scale, SUS) [38] will be completed after finishing module 1 and module 4. The research team will record the usage that each participant makes of the platform: the number of times it was accessed, modules completed, and number of tasks delivered. Two external professionals, one in each province and unrelated to the research group, will be in charge of monitoring the intervention, providing feedback to the participants, and making weekly contact with participants via their preferred means (telephone call, text message, or email); this contact will be used to motivate participants to complete the intervention. Both professionals will previously have been trained in the contents and procedures of the MINDxYOU program by the research team. Once the intervention is complete, focus groups will be held in the provinces of Zaragoza and Malaga to qualitatively assess the acceptance and suitability of the intervention, and interviews will be conducted with managers and directors from each site to evaluate different aspects regarding implementation of the program.

**Table 1:** Brief outline of the MINDxYOU program

Module	Main contents	Main practices
0. Welcome module	This module will introduce participants to the platform and present the main aims of the intervention	Participants will be given the opportunity to share their feelings and needs, and will receive feedback from the research team
1. Mindfulness	The concept of mindfulness will be explained, together with its applications for stress reduction and the promotion of good mental health	Body scan, sitting meditation, three-minute breathing meditation
2. Compassion	The concept of compassion will be explained, together with its applications for stress reduction and the promotion of good mental health	Compassionate breath meditation, compassionate body scan, safe place meditation
3. Acceptance	The concept of acceptance will be explained, together with its applications for stress reduction and the promotion of good mental health	Meditation focused on acceptance, identification of personal values
4. Healthy lifestyle	Instructions on how to apply the concepts of mindfulness, compassion, and acceptance to the main aspects of a healthy lifestyle: physical activity, diet, sleep, and socialization	Mindfulness and compassion exercises applied to physical activity, diet, sleep, and socialization

### Intervention

The MINDxYOU online program is based on the principles of “third wave” psychotherapies, such as the promotion of wellbeing through the practice of mindfulness, compassion, acceptance, and spirituality [39]. The effectiveness of “third wave” interventions has been widely contrasted empirically, also in the case of health professionals [16], and the MINDxYOU program was designed based on the experience of the research group on previous projects, such as the Attachment-Based Compassion Therapy [40] and Smiling is Fun [41] online programs, and also considering the results of the HEROES [42] and the PSY-COVID projects, the latter being an international research project that evaluated the psychosocial impact of the COVID-19 pandemic on both the general population and specific populations (healthcare personnel, university students, etc.).

MINDxYOU is a self-guided online program particularly designed for healthcare workers in order to both monitor and support the individual’s mental health. This program will aid healthcare professionals to deal with their daily, work-related stress through the practice of mindfulness, compassion, and acceptance exercises, along with indications on how to apply these concepts to basic aspects of a healthy lifestyle (i.e., physical activity, sleep hygiene, balanced diet, and socialization).

The program is self-administered and will be delivered over the Internet and available via smartphone, tablet, and personal computer. During the first 8 weeks, there will be minimum support provided by an external professional unrelated to the research group, who will make weekly contact with participants via their preferred means (i.e., telephone, text message, email) to promote adherence to the intervention, encouraging the participant to keep on using the program and strengthening their commitment to the exercises. However, this contact will neither be used to add new educational content nor for assessment purposes, which will be entirely covered by the online platform with online links to the questionnaire(s) they need to complete; nor will it be used for counseling purposes.

The intervention begins with a welcome module that introduces the main aims and procedures of the platform. Participants are invited to share a written testimony of their main stressors in their workplace, and their feelings and personal needs. The external professional will read each testimony and respond to each one by offering a rationale for why the intervention can be beneficial for their case, enhancing motivation to complete the intervention. The testimonies will be analyzed and used to improve the contents of the intervention wherever possible.

After the welcome module, the program will be structured into 4 modules, each dedicated to a specific topic (see Table 1). Every module will contain animated videos with explanations of the aims and contents of the topic to be addressed, along with some examples of exercises that participants will then be able to practice on their own. Written material will also be provided to give participants a deeper understanding of the contents of the module, and audios with guided practices will be included for each exercise. PDF files and audios will be available for participants to download and save for use in the future or for practice outside of the web platform. Practice logs will also be available, and participants will be able to complete them on the online platform to enable the research team to check the completion of tasks. It is recommended that a period of 2 weeks be dedicated to each module, although participants will be free to adapt this to their needs. The first module, on mindfulness, will introduce most of the basic concepts of the intervention, and these will also be taken up in the following modules. The last module will focus on how to apply the concepts and practices of mindfulness and compassion concepts to key aspects related to a healthy lifestyle (i.e., physical activity, diet, sleep, and socialization). The estimated time required to complete the whole program is 8 weeks; after week 8, participants will no longer be contacted by the member of the research team. However, the program will continue to be available for use indefinitely.

### Assessment plan

All potential candidates will undergo an initial screening assessment conducted by a member of the research team who is a certified psychiatrist or clinical/health psychologist; this assessment will include MINI v7.0.2 to identify psychiatric disorders. Later, a baseline evaluation will be conducted, including an ad hoc sociodemographic questionnaire, primary and secondary outcomes, process variables, and cost-utility measures. After the baseline evaluation, the participants will be assessed again every 8 weeks. During the intervention, the participants will complete the SUS twice.

#### Diagnostic interview: Mini International Neuropsychiatric Interview (MINI) Version 7.0.2

MINI v7.0.2 [43] is a brief structured diagnostic interview for DSM-5 psychiatric disorders. It assesses the 17 most common disorders in mental health, those considered the most important to identify in clinical and research settings: major depressive episode, major depressive disorder, suicidal tendencies, suicidal behavior disorder, maniac episode, hypomanic episode, bipolar disorder type I, bipolar disorder with psychotic traits, bipolar disorder type II, other bipolar disorder, panic disorder, agoraphobia, social anxiety disorder, obsessive–compulsive disorder, post-traumatic stress disorder, alcohol abuse disorder, and substance abuse disorder. It takes approximately 15 min to administer, and the present study will use the Spanish version to verify that the participants do not present with any severe conditions. MINI has shown excellent psychometric properties, including high interrater reliability (*k* = 0.90 or higher) [43].

#### Primary outcome: Perceived Stress Scale (PSS)

PSS [37] consists of 10 items in which participants are asked to rate how unpredictable, uncontrollable, and overloaded they have found their life over the past month on a 5-point Likert-type scale (e.g., “how often have you felt that you were unable to control the important things in your life?”). Scores range between 0 and 40, and higher scores reflect higher levels of perceived stress. PSS was validated in Spanish [44], and this version presented good psychometric properties, including internal consistency (α = 0.81), test–retest reliability (*r* = 0.77), and sensitivity to change.

#### Secondary outcomes

The *Patient Health Questionnaire* (PHQ-9) [45] is a 9-item scale aimed at screening for depression in primary care and other medical settings. It assesses the frequency of depressive symptoms during the last 2 weeks using a scale between 0 (not at all) and 3 (nearly every day). The total score ranges between 0 and 27, with higher scores indicating higher severity of depression. PHQ-9 has presented excellent psychometric properties, including reliability, validity, and responsiveness [46].

The *Generalized Anxiety Disorder-*7 (GAD-7) [47] is a 7-item, self-report measure to assess the intensity of anxiety symptoms. It refers to the period of the past 2 weeks; each item is scored on a 4-point Likert-type scale, resulting in a total score that can range between 0 and 21, with higher values reflecting more severe anxiety symptoms. Good psychometric properties have been reported for GAD-7 in different populations, also for the Spanish version [48].

The *Brief Symptoms Inventory-18* (BSI-18) [49] is an adapted version of the original 53-item, self-report questionnaire [50] that is designed to offer rapid screening for the symptoms of psychological disorders. Each item is scored on a 5-point Likert-type scale, reflecting on the past 7 days. Scores on the 18 items are summarized on the Global Severity Index (GSI). The BSI-18 also includes three symptom scales: Somatization, Depression, and Anxiety, each comprising six items. The Spanish version [51] shows reliable and valid psychometric properties.

#### Process variables

The *Connor-Davidson Resilience Scale* (CD-RISC) [52] is a 10-item, self-report measure addressed at assessing resilience. Each item is scored on a 5-point Likert scale, and the total score, which ranges between 1 and 5, is calculated by averaging the scores of the items; higher scores indicate higher levels of resilience. The questionnaire has shown good psychometric properties in people with anxiety or stress-related disorders. The Spanish version [53] also presented good internal consistency (*α* = 0.86) and test–retest reliability (*r* = 0.87).

The *Five Facets of Mindfulness Questionnaire -15 item version* (FFMQ-15) [54] is an adaptation of the original 39-item FFMQ, a questionnaire measuring the five facets of mindfulness: *observing* (i.e., noticing internal and external experiences such as sensations, thoughts, or emotions), *describing* (i.e., labeling internal experiences with words), *acting with awareness* (i.e., focusing on one’s activities in the present moment as opposed to behaving automatically), *nonjudging of inner experience* (i.e., taking a non-evaluative stance toward thoughts and feelings), and *non-reacting to inner experience* (i.e., allowing thoughts and feelings to come and go, without getting caught up by them). Each subscale of FFMQ-15 includes 3 items, scored on a Likert-type scale (1–5). A score for each subscale, ranging between 5 and 15, can be computed by summing the items, where higher scores indicate higher levels of the mindfulness facet. FFMQ-15 presents good psychometric properties, similar to those of the original questionnaire, and the Spanish version also presented good internal consistency for every subscale [55].

The *Sussex-Oxford Compassion Scales* (SOCS) [56] consist of two self-report measures designed to evaluate self-compassion and other-compassion. Each measure consists of 20 items that are answered on a 5-point Likert-type scale. A total score of each scale can be calculated by summing the scores of the items; each total score ranges between 20 and 100, with higher scores indicating higher levels of compassion. The psychometric properties of the SOCS have proved to be good [56], and the Spanish adaptation is currently being validated by our research group.

The *Acceptance and Action Questionnaire-II* (AAQ-II) [57] is a common measure for assessing experiential avoidance, a key element of “third wave” psychotherapies that refers to the tendency to avoid thoughts, feelings, memories, sensations, and other internal experiences, and which is very commonly associated with worse mental health outcomes, as opposed to psychological flexibility. The scale presents 7 items scored on a 7-point Likert-type scale, and the total score is calculated by summing all the items. Higher scores mean higher experiential avoidance. AAQ-II shows good psychometric properties, including internal consistency (α = 0.84) and test–retest reliability (*r* = 0.79). The Spanish version has presented similar psychometric qualities [58].

#### Cost-utility variables

The five-level version of the *EuroQol five-dimensional classification system* (EQ-5D) [59] is a widely used health-related quality of life measure. First, participants are asked to report the severity (1 = no problems, 2 = mild, 3 = moderate, 4 = severe, 5 = extreme) of the problems they may present on the day of reporting with regard to each of the following five domains: mobility, self-care, usual activities, pain, and anxiety/depression. The combination of the answers given to these domains results in 3,125 different health states. The utility scores are obtained from the EQ-5D classification system and are used to rate patients’ health-related quality of life, which normally range between 0 (although it is possible to present negative scores) and 1 (i.e., “perfect health”). These utility values will be calculated using the Spanish tariffs of EQ-5D-5L [60]. EQ-5D utility values will be used to estimate quality-adjusted life-years (QALYs), a common measure to assess the outcomes associated with different treatments, both in terms of patients’ quality of life and survival [61]. EQ-5D also asks participants to use a visual analog scale (EQ-VAS) to record their current overall health status, ranging between 0 (worst imaginable health) and 100 (best imaginable health).

The *Client Service Receipt Inventory* (CSRI) [62] is one of the most widely used resource-use measurement tools; it describes and measures service utilization patterns as a basis for estimating associated costs across healthcare, social care, and community settings. In the present study, the Spanish version [63] will be used to collect retrospective data (last 2 months) on medication and service use. For medication intake, patients will be asked to bring their daily medication prescriptions, and the following information will be recorded: drug name, dosage, total number of prescription days, and daily dosage consumed. With regard to service use, patients will be asked about total visits to emergency departments, total days of general inpatient hospital admissions, number of diagnostic tests administered, and total visits to primary care physician, nurse, social worker, psychologist, psychiatrist, group psychotherapy, and other community healthcare professionals, specifying in each case if these services were provided by the public or by the private sector.

#### Implementation outcomes

In accordance with the Consolidated Framework for Implementation Research (CFIR) model, and taking into consideration the guidelines by Proctor et al. [64] and recommendations for implementation studies of online interventions by Hermes et al. [65], the present study will assess the following outcomes for the implementation process: acceptability, adoption, appropriateness, feasibility, fidelity, penetration, sustainability, and implementation costs, which will be measured along with the cost-utility variables. Treatment expectancies will be assessed before commencing the intervention, and participants will again be asked the same questions after completing the last module to assess their opinion [66]. In addition, focus groups will be held with the key players involved in the study in each province (i.e., Zaragoza and Malaga) once the intervention is complete (posttreatment) to qualitatively evaluate different domains related to the implementation.

The first of these, a*cceptability*, defined as the perception among implementation stakeholders that the intervention is useful or satisfactory, will be measured using the following measures:

- The *System Usability Scale* (SUS) [67], which is a 10-item questionnaire to measure the usability which is qualitatively related to the quality and acceptability of the intervention [68]. Usability is defined as the ease of use perceived by the users of the implemented technology. Use will be made of the Spanish version of SUS, which has presented good internal consistency (α = 0.81) [69].
- The *Attitudes towards Psychological Online Interventions* (APOI) [70], which includes 16 items to assess attitudes toward online interventions. APOI explores four dimensions; “Skepticism and Perception of Risks,” “Confidence in Effectiveness,” “Technologization Threat,” and “Anonymity Benefits”. APOI has shown good psychometric properties with acceptable to good internal consistency (α = 0.77) [70].
- The *Client Satisfaction Questionnaire adapted to Internet-based interventions* (CSQ-I) [71] is an 8-item, 4-point Likert scale questionnaire that assesses the general satisfaction of participants regarding the received intervention. The total score ranges between 8 and 32. The scale has shown excellent internal consistency (α = 0.93) [72].

Another construct that will be assessed in this regard is *appropriateness*, which is defined as perceived fit, relevance, or compatibility of the evidence-based practice for a given practice setting. We will use the *Intervention Appropriateness Measure* (IAM) [73], which includes 4 items designed to measure appropriateness of the intervention. The scale has shown good psychometric properties with high levels of internal consistency (α = 0.85 to 0.91) and test–retest reliability coefficients (*r* = 0.73 to 0.88).

The third domain that will be assessed in terms of implementation is *adoption*, defined as the intention, initial decision, or action to try or employ an evidence-based practice. In our study, adoption will be measured by the number of participants who agreed to use the program (considering the total number that were invited to use it) and actually accessed the online program. We will gather this information from the passive data collected by the online platform.

The *feasibility* of the intervention will also be studied. This is defined as the extent to which a new treatment can be successfully used or carried out within a given agency or setting. Feasibility will be measured with passive data gathered by the online platform regarding use of the program, specifically the frequency of use during the study. Use will also be made of the *Feasibility of Intervention Measure* (FIM) [73], which includes 4 items designed to measure the feasibility of the intervention. The scale has shown good psychometric properties with high levels of internal consistency (α = 0.85 to 0.91) and test–retest reliability coefficients (*r* = 0.73 to 0.88).

The fifth domain of implementation that will be analyzed is *fidelity*. In the context of online interventions, fidelity is defined as expected clinically meaningful use. This dimension will be measured with passive data collected by the online platform, specifically the number of modules and tasks completed. *Penetration*, in turn, will be measured considering the number of participants who agreed to use the intervention and completed at least 4 of the 5 modules of the program. Finally, *sustainability*, defined by the extent to which a new intervention is maintained, will be evaluated during the interviews and/or focus groups with the managers or directors of the different sites.

### Data analysis

#### Effectiveness analysis

Both intention-to-treat analysis and per-protocol analyses (i.e., those participants who complete at least 4 of the 5 modules of the intervention) will be performed. The analyses will include the description and elementary head-to-head comparisons between both phases (i.e., control vs. intervention). Specifically, the variables for each of the phases will be described by using descriptive statistics (means and 95% confidence intervals in the case of quantitative variables with normal distribution, medians, and interquartile range in the case of quantitative variables with non-normal distribution). To confirm the main hypothesis, all of the effectiveness variables (i.e., PSS and secondary outcomes) will be compared between the intervention and control phases. A multilevel regression analysis will be performed including the cluster as a random effect, and multivariate multilevel regression analysis will be performed to adjust for possible confounders. The magnitude of the effect of the improvement and the Number Needed to Treat (NNT) will also be reported, using the cut-off points already established in the primary outcome as criteria for considering whether a participant is a responder.

#### Economic evaluation

The economic evaluation will be carried out following the recommendations of the Spanish guidelines for the economic evaluation of health technologies [74]. A societal perspective will be adopted, considering both direct and indirect costs. The cost-utility analyses will consider the effect of the intervention on the QALYs, and the results will be expressed in terms of the incremental cost-utility ratio (ICUR), calculated by dividing the difference in total costs between the intervention phase and the control phase by the difference in QALYs between both phases. A cost-effectiveness analysis will be also conducted, in this case considering the effect of the intervention on perceived stress (PSS). These results will be expressed in terms of the incremental cost-effectiveness ratio (ICER), calculated by dividing the difference in total costs between the treatment phase and the control phase by the difference in PSS scores between both phases.

#### Implementation analysis

In order to perform the analysis related to the implementation process of the intervention, different outcomes will be considered according to the adopted model [64, 65]. The analysis of the passive data consists of objective and direct counts (e.g., logins, frequency of use, modules completed, task completed, etc.). The quantitative data extracted from the questionnaires will be analyzed according to the appropriate methodology in each case. Some data will be collected qualitatively through interviews with the different stakeholders.

## Ethics and dissemination

All procedures performed in this study will adhere to the 1964 Declaration of Helsinki and its most recent amendments (7th revision, adopted by the 64th World Medical Association General Assembly, Fortaleza, Brazil). Signed informed consent will be obtained from all participants, who will not receive any compensation, once they have been informed of the study procedures, potential risks, and their right to withdraw at any time from the study. The Research Ethics Committee of the Autonomous Community of Aragon (CEICA) and the Ethics and Research Committee of Northeast Malaga evaluated and approved the study protocol in July 2022 (PI22/341). Any important protocol modifications (e.g., changes to eligibility criteria, outcomes, analyses) will need to be approved by the ethics committees. To guarantee the confidentiality of the information, all the data collected by the platform will comply with the provisions of the Spanish Data Protection and Digital Rights Act 3/2018 (LOPD), which adapts Spanish legislation to the European Union’s General Data Protection Regulation (GRDP). A specific plan to guarantee maximum security of all collected data will be implemented for this project, and an assessment will be made of any possible risks and impacts on the information flow. The technology platforms will be separated into two completely independent systems, which will access two also independent databases. This will provide complete disaggregation of patient data, i.e., users’ personal data will be separated from their clinical record, multiplying by 2 all the security measures protecting end users. The data will remain separate on 2 different servers, with clinical data hosted on server 1 and personal data on server 2.

Once the study is complete, we will publish our results in international peer-reviewed biomedical journals and present them at national and international conferences. The implementation study will be conducted and reported according to the Standards for Reporting Implementation Studies (StaRI) Statement, indicated for reporting implementation studies focusing on enhancing the adoption and sustainability of the intervention [75]. A report will also be sent to the Instituto de Salud Carlos III (main funding body). The lead researcher will organize an end-of-study knowledge translation seminar. The main aim of this activity will be to share the study findings with stakeholders in order to discuss how to maximize uptake of the findings and to determine future research directions.

## Discussion

Healthcare workers are in need of effective interventions to reduce their stress levels, which have been very significantly affected by the COVID-19 pandemic, in order to bring about a reduction in their risk of developing mental health disorders and, consequently, enhancement of their quality of life and job performance. The pandemic has helped to raise the visibility of healthcare workers as a risk group for developing mental health problems, which has facilitated the proliferation of different interventions in the recent years to address the needs of this population.

In this regard, online interventions have become particularly popular, both during and after the pandemic, as they offer a solution when face-to-face treatments are not available or recommended. Different programs have been adapted to the online format, mainly those based on classic CBT and “third wave” psychotherapies [20, 23–30]. Both types of interventions have been tested, with generally positive effects in stress reduction, although the effect sizes in many cases were small, which indicates that there is still room for improvement. The problem with some of these interventions might be that they deal with stress in a more generic way, rather than focusing on the kind of stressors that healthcare workers face on a daily basis. In this regard, a systematic review [76] suggested that the next step when developing online interventions targeting healthcare professionals was to adjust them to the particular needs of this population; therefore, an intervention that considers this aspect could generate both a higher degree of adherence and greater impact in terms of stress reduction.

MINDxYOU is a self-guided, Internet-delivered program that has been designed following the path drawn by previous studies offering online interventions to deal with different psychopathological symptoms (e.g., depression, anxiety, stress) [25, 26, 40, 41] and, using the feedback provided by different healthcare workers (i.e., doctors, nurses, psychologists), it adds a focus customized to the needs of these professionals, with examples that relate to common problems in their daily lives. The program offers different modules that enable the practice mindfulness, acceptance, and compassion, which constitute the core of the intervention, and a final module focusing on how to apply such skills to the main aspects of a healthy lifestyle (i.e., diet, sleep, exercise, socialization). This is justified by the importance of such aspects in mental health, and considering that many healthcare professionals have neglected their personal needs owing to their high levels of stress during the pandemic [2]. On the whole, MINDxYOU aims to cover the main needs of healthcare workers with regard to their stress management, and the present study will test whether the program is both effective and cost-effective.

Furthermore, the problem with many psychological interventions is the gap between research and practice. This means that, despite being proven effective, interventions generally take quite a number of years to be applied in the real-world practice [31]. Thus, implementation science focuses on the development of studies to reduce the know-do gap, focusing on the barriers to and facilitators for the implementation process [77]. Based on previous RCTs, we expect to adapt the intervention according to users’ experience and opinions [78, 79]. The implementation study will be conducted in order to assess the impact of the intervention on participants’ stress levels, the direct and indirect costs of the intervention, and the different factors influencing the implementation process: acceptability, appropriateness, adoption, feasibility, fidelity, penetration and sustainability.

If our hypotheses are confirmed, MINDxYOU will turn out to be both effective and cost-effective, and our results will provide information on the potential use of information and communications technologies (ICTs) related to the cost-effectiveness of Internet-based psychological interventions. Moreover, the study will show how implementation studies are useful to establish the framework with which to deal with the barriers to online psychotherapies and promote their implementation. In summary, this study could make a significant contribution to promote the use of online psychotherapy for reducing healthcare workers’ stress and reduce the gap between research and practice. 

## Data Availability

Upon completion of the study, the datasets generated and/or analyzed during the current study will be made available to researchers on request to the corresponding authors: Adrián Pérez-Aranda (aparanda@iisaragon.es) and Alberto Barceló-Soler (abarcelosoler@hotmail.com). No manuscripts related to this study have yet been published or submitted to any journal.

## Abbreviations

AAQ: Acceptance and Action Questionnaire
ACT: Acceptance and Commitment Therapy
APOI: Attitudes towards Psychological Online Interventions
BSI: Brief Symptom Inventory
CBT: Cognitive-Behavioral Therapy
CD-RISC: Connor-Davidson Resilience Scale
CFIR: Consolidates Framework for Implementation Research
CSQ-I: Client Satisfaction Questionnaire adapted to Internet-Based interventions
CSRI: Client Service Receipt Inventory
EQ-5D: EuroQoL 5 Dimensions
FFMQ: Five Facet Mindfulness Questionnaire
FIM: Feasibility of Intervention Measure
GAD: Generalized Anxiety Disorder
IAM: Intervention Appropriateness Measure
ICER: Incremental Cost-Effectiveness Ratio
ICT: Information and communications technology
ICUR: Incremental Cost-Utility Ratio
MBI: Mindfulness-Based Interventions
NNT: Number Needed to Treat
PC: Primary Care
PHQ: Patient Health Questionnaire
PSS: Perceived Stress Scale
QALY: Quality-Adjusted Life Year
RCT: Randomized Controlled Trial
SOCS: Sussex-Oxford Compassion Scales
SUS: System Usability Scale
SW: Stepped Wedge
WHO: World Health Organization
